# Isolated Gallbladder Injury Secondary to Blunt Abdominal Trauma

**DOI:** 10.7759/cureus.15337

**Published:** 2021-05-30

**Authors:** Mohamed Abouelazayem, Raluca Belchita, Dimitrios Tsironis

**Affiliations:** 1 General Surgery, St George’s University Hospitals NHS Foundation Trust, London, GBR; 2 General Surgery, St George's University Hospitals NHS Foundation Trust, London, GBR; 3 Upper Gastrointestinal Surgery, St George's University Hospitals NHS Foundation Trust, London, GBR

**Keywords:** gall bladder, isolated gallbladder injury, blunt abdominal trauma, gall bladder perforation, gall bladder trauma

## Abstract

A 60-year-old male was admitted to our major level 1 trauma centre following a fall from the fourth storey of a car park and landing initially on his feet on concrete. The primary survey was unremarkable apart from abdominal pain and localised peritonism in the right upper quadrant and lower lumbar midline pain. The secondary survey revealed bilateral complex calcaneal fractures, multiple vertebral fractures and sternal fracture. A trauma CT scan showed pericholecystic fluid and described by the radiology team either as cholecystitis picture or possible disruption of the gallbladder wall. Based on the patient’s stable presentation, the decision was made for a diagnostic laparoscopy to explore possible gallbladder injury and other concomitant injuries.

Operative findings showed free bile in the right upper quadrant and right paracolic gutter and small amount of blood. The gallbladder did not have an obvious site of perforation but had a necrotic appearance. No further injuries identified laparoscopically after checking small and large bowel, and since no obvious perforation was identified, the decision was made to convert to laparotomy and duodenal exploration. On laparotomy, there was no evidence of duodenal or pancreatic injury on Kocher’s manoeuvre and ligament of Trietz mobilisation. The gall bladder wall was stained and leaking bile, therefore a standard retrograde cholecystectomy was performed. No further intra-abdominal injuries were identified during the laparotomy. The patient made an unremarkable recovery. He was discharged home with physiotherapy for rehabilitation.

We recommend a diagnostic laparoscopy and cholecystectomy for such injuries with a low threshold for duodenal exploration (Kocherization) if the perforation site is not obvious based on the high incidence of concomitant duodenal injuries.

## Introduction

Gallbladder injuries from blunt abdominal trauma are rare as the gallbladder is embedded in the liver parenchyma and protected by the ribcage. The blunt forces required to injure the gallbladder in this protected area most likely will cause other serious intra-abdominal injuries. More than 98% incidence of concomitant intra-abdominal pathology is described in the literature. It is usually these aforementioned injuries that determine the patient’s management and prognosis.

Initial clinical signs and symptoms of gallbladder injuries are often non-specific, before a period of clinical deterioration secondary to bilious peritonitis. As a consequence, a delay in the diagnosis of isolated gallbladder injuries is not uncommon.

The purpose of presenting this case is to increase the clinician awareness of the possibility of gallbladder perforation after blunt abdominal trauma in order to focus diagnostic suspicion and enhance early detection.

## Case presentation

A 60-year-old male was admitted to our major trauma centre following a fall from the fourth storey of a car park and landing initially on his feet.

He had a background of chronic obstructive pulmonary disease, peripheral vascular disease, hypertension, depression, osteoporosis, and previous alcohol dependence that had stopped 20 years ago. On scene assessment by paramedics, he was found to be haemodynamically normal and stable (temperature 36.8°C, heart rate 94 beats per minute, blood pressure of 110/70 mmHg, oxygen saturation of 98% on room air) with a Glasgow Coma Scale of 15; therefore, he was transferred to the hospital with road ambulance. Standard ATLS (advanced trauma life support) protocol was followed on hospital assessment.

The primary survey was unremarkable apart from abdominal pain and localised peritonism in the right upper quadrant and lower lumbar midline pain. Secondary survey revealed bilateral complex calcaneal fractures, for which he required surgery later on, multiple vertebral fractures, and sternal fracture, which were managed conservatively.

On admission, the blood tests showed elevated WBC (white cell count) of 16.3 109/L (with neutrophilia 14.7 109/L), raised alanine aminotransferase (ALT) of 106 U/L and Gamma-glutamyl transferase (GGT) of 100 U/L. The rest of the bloods, including routine biochemistry and coagulation profile, were all within normal limits. No haematuria was present on urinalysis.

A trauma CT scan was obtained, which showed pericholecystic fluid and described by the radiology team either as cholecystitis picture or possible disruption of the gallbladder. In the context of trauma, isolated intra-abdominal injury of the gallbladder should be considered, although this is uncommon. The patient denied any previous right upper quadrant pain or history suspicious of cholecystitis. CT head, neck, thorax, and lower limbs were consistent with the secondary survey findings.

Based on the patient’s stable presentation, a decision was made for diagnostic laparoscopy to explore possible gallbladder injury and other concomitant injuries. The procedure took place on the same day.

**Figure 1:**
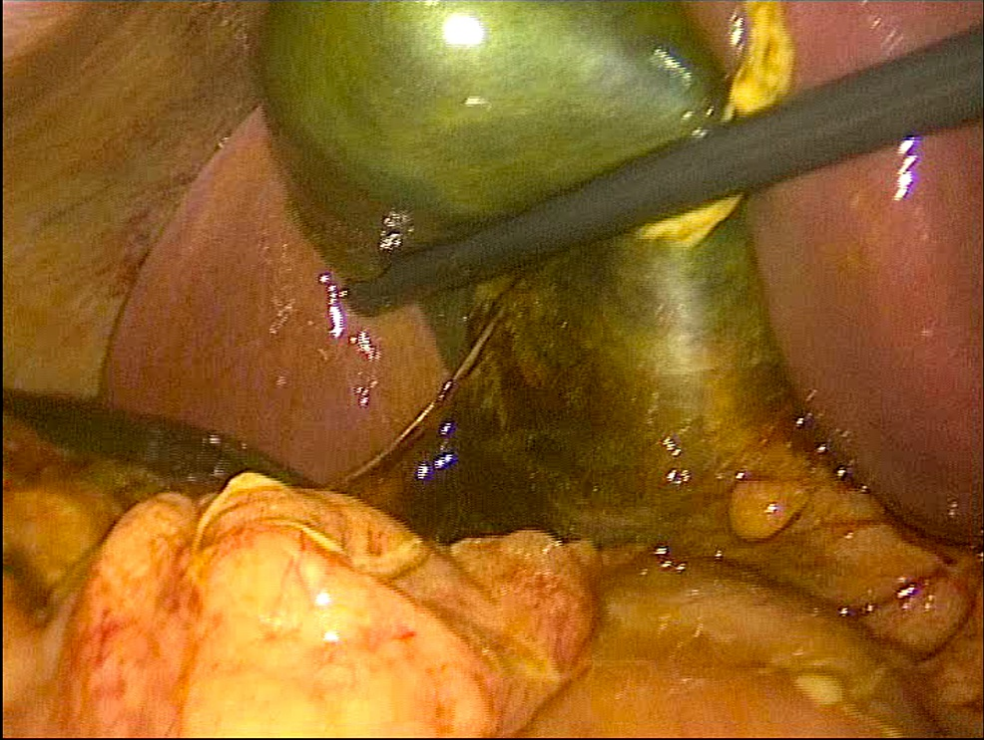
Necrotic appearance of gallbladder without obvious site of perforation

Operative findings showed free bile in the right upper quadrant and right paracolic gutter and a small amount of blood. The gallbladder did not have an obvious site of perforation but had a necrotic appearance (Figure 1)

There was no liver injury identified to account for bile spillage.

The stomach, lesser sac, and small and large bowel were assessed laparoscopically, and no injuries were identified. Moreover, there was no hepatoduodenal ligament injury identified on assessment for possible extrahepatic biliary injures.

**Figure 2:**
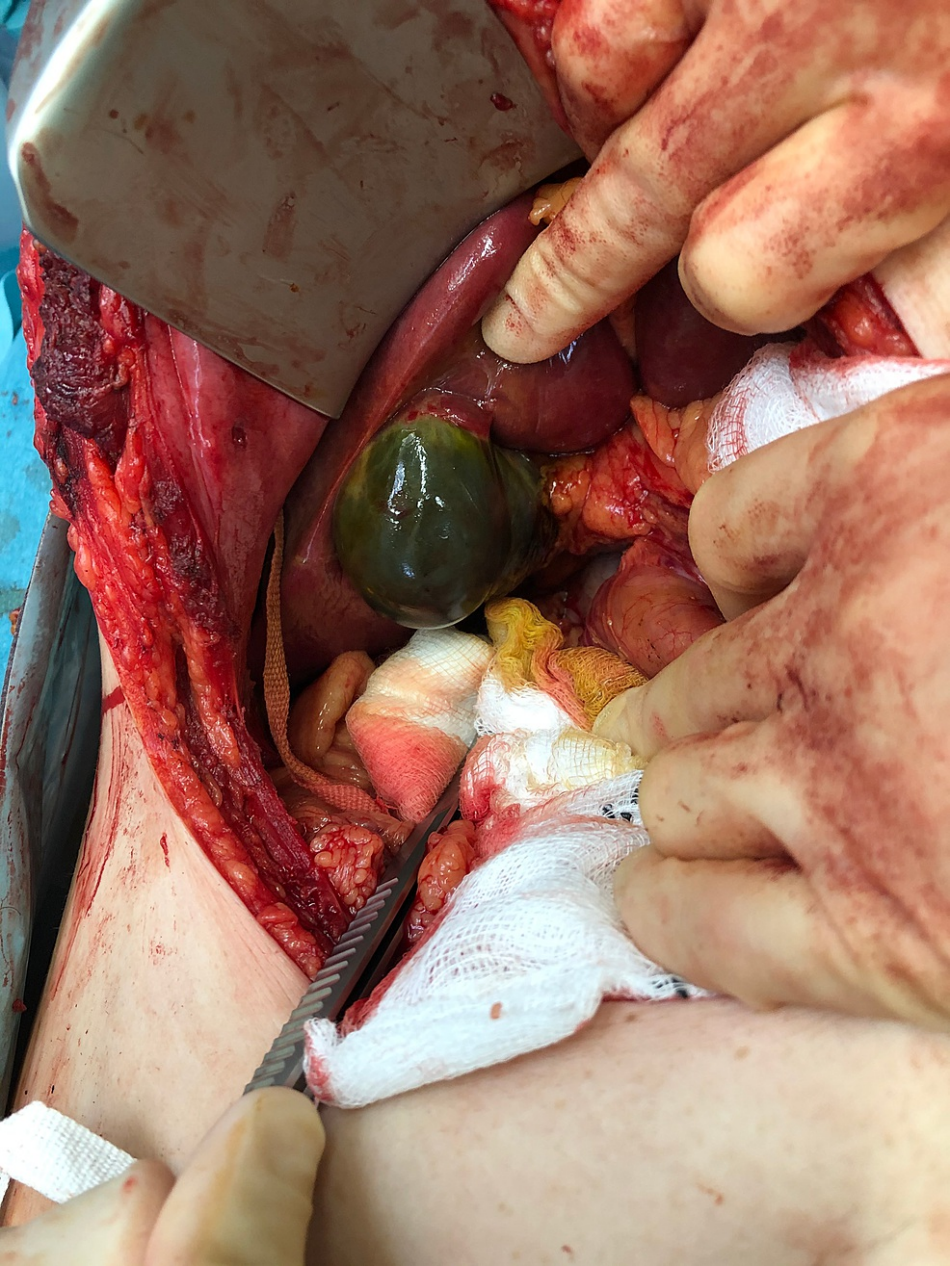
Gallbladder wall stained and leaking bile

**Figure 3:**
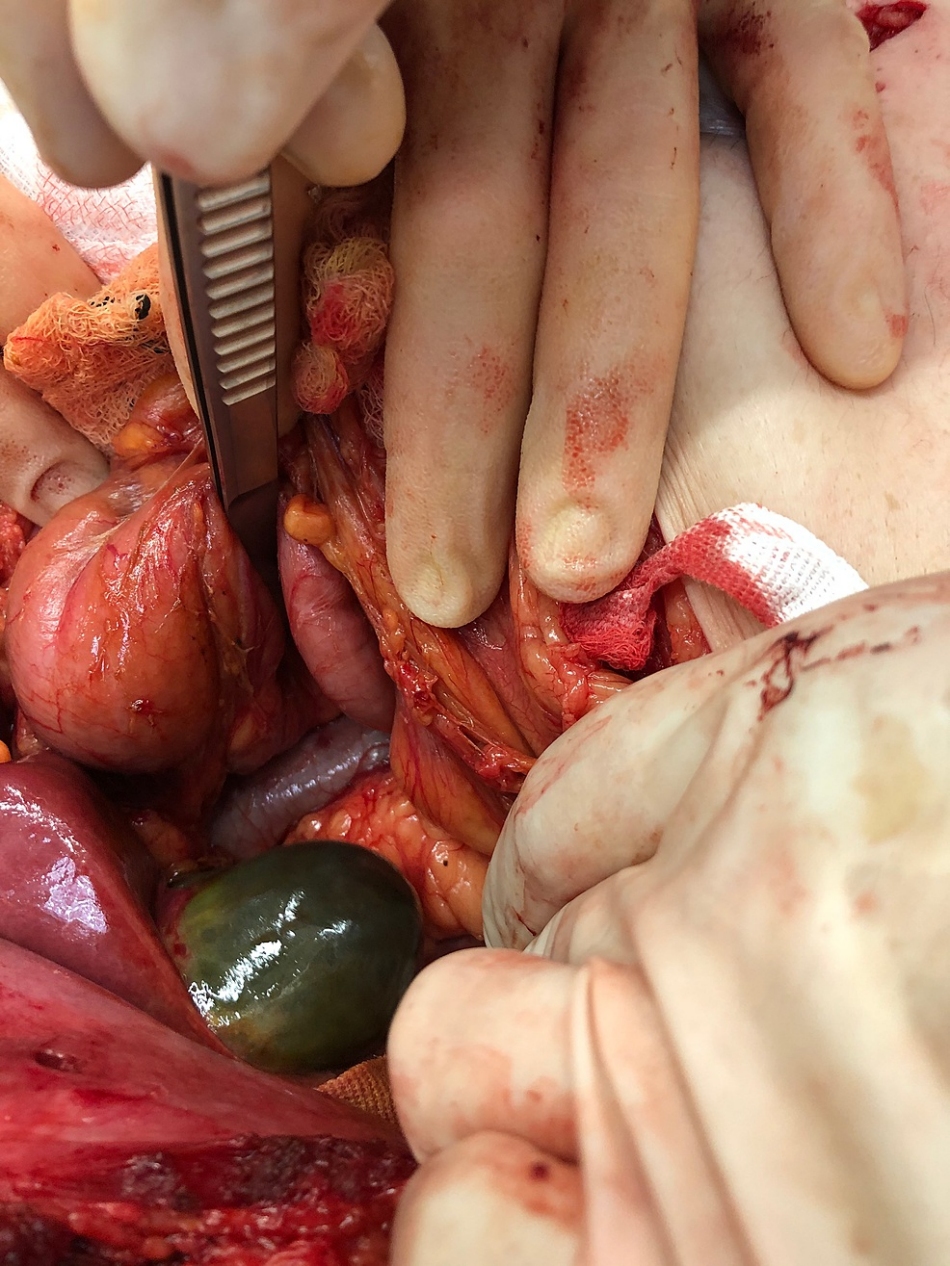
Necrotic and bile-stained appearance of the gallbladder

Since no obvious perforation was identified laparoscopically, a decision was made to convert to laparotomy and duodenal exploration. On laparotomy, there was no evidence of duodenal or pancreatic injury on Kocher’s manoeuvre and ligament of Trietz mobilisation. Gallbladder wall was stained and leaking bile (deceleration injury) (Figures 2,3), therefore, a standard retrograde cholecystectomy was performed. 

No further intraabdominal injuries were identified during the laparotomy. Two abdominal drains were inserted at the end of procedure, one in the pelvis and one in the Morrison’s pouch, and removed during postoperative days without any signs of bile or enteric fluid.

The patient was transferred intubated postoperatively to the ITU (intensive therapy unit) and was extubated the following day.

The patient made an unremarkable recovery from general surgery point of view. He had an operation under the orthopaedic team for the management of calcaneal fractures. He was discharged home with physiotherapy for rehabilitation mainly due to calcaneal fractures.

Gross pathology of the gallbladder showed a shiny and smooth serosa. The thickness of the gallbladder wall was <4 mm and one small stone was found measuring 7 mm in maximum diameter. Microscopically, the pieces of the gallbladder wall showed features of cholecystitis and necrosis.

## Discussion

Isolated gallbladder injury/rupture from blunt abdominal trauma is seldom mentioned in literature due to its rarity. The incidence of injury to the gallbladder from blunt abdominal trauma has been reported as 0.5±0.6% of all the intra-abdominal injuries [1].

Liver injury is especially likely: (83% to 91%) of patients with gallbladder injuries; duodenum and spleen injuries in up to 54% of patients [2]. Our patient had no other intra-abdominal injuries.

Gallbladder injuries can range from contusion, laceration, to partial or complete avulsion. Contusion may pass undiagnosed due to lack of acute symptoms [1]. Our case represents a deceleration injury which resulted in bile leak from the wall of gallbladder and histology appearances of acute necrosis of the gallbladder wall. There was no avulsion of gallbladder from liver bed intraoperatively.

The mechanism of gallbladder injury in blunt trauma involves compression and shearing forces. The factors which predispose the gallbladder to perforation in blunt trauma include a thin-walled normal gallbladder, gallbladder distension, and alcohol ingestion, which increase the sphincter of the Oddi tone and raise the biliary tract pressure [1].

Presentation in these cases can be delayed. Leakage of sterile, non-infected bile into the peritoneum will lead to picture of biliary peritonitis. The use of imaging modalities such as CT or sonography are useful for an early diagnosis of gallbladder perforation [3]. A previous case report mentioned a patient who was treated conservatively for possible gallbladder injury without CT imaging on the basis of clinical findings and ultrasound, was discharged home and represented two days later with biliary peritonitis. This highlights the importance of high degree of suspicion and early CT imaging in these cases with low threshold for diagnostic laparoscopy [4]. CT scan findings can include defect in gallbladder wall, pericholecystic fluid collection, or stranding in omentum or mesentery.

In a similar case, the authors recommended laparoscopic cholecystectomy as the treatment for isolated gallbladder injuries. They also recommended a high index of suspicion for other associated injuries and conversion to exploratory laparotomy in cases with uncertainty in diagnosis [5].

Duodenal injuries from blunt abdominal trauma also has been reported in literature with an incidence of 11.2% to 26%. Duodenal injuries can be missed, especially posterior wall perforations. A previous case report mentioned that a posterior wall perforation was missed on the first laparotomy since there was no obvious anterior wall perforation [6].

In our case, there was no clear perforation on the gallbladder wall during the diagnostic laparoscopy or other visceral injuries to account for bile leak, so we decided to fully explore the duodenum (Kocherization) for possible associated duodenal injury.

## Conclusions

Gallbladder rupture in blunt abdominal trauma is rare. Isolated gallbladder injury is even more unusual. A high degree of suspicion based on clinical findings with early imaging taking into account the mechanism of injury is recommended to avoid delayed diagnosis. We recommend a diagnostic laparoscopy and cholecystectomy for such injuries with a low threshold for duodenal exploration, especially if a perforation site is not obvious, based on the high incidence of concomitant duodenal injuries.
